# Inhibition of Poly ADP-Ribose Glycohydrolase Sensitizes Ovarian Cancer Cells to Poly ADP-Ribose Polymerase Inhibitors and Platinum Agents

**DOI:** 10.3389/fonc.2021.745981

**Published:** 2021-10-27

**Authors:** Emad Matanes, Vanessa M. López-Ozuna, David Octeau, Tahira Baloch, Florentin Racovitan, Amandeep Kaur Dhillon, Roy Kessous, Oded Raban, Liron Kogan, Shannon Salvador, Susie Lau, Walter H. Gotlieb, Amber Yasmeen

**Affiliations:** ^1^ Division of Gynecologic Oncology, Jewish General Hospital, Montreal, QC, Canada; ^2^ Segal Cancer Center, Lady Davis Institute of Medical Research, McGill University, Montreal, QC, Canada

**Keywords:** ovarian cancer, targeted therapies, homologous recombination, poly (ADP-ribose) glycohydrolase (PARG), poly (ADP-ribose) polymerase (PARP) inhibitors

## Abstract

**Background:**

Poly ADP-ribose glycohydrolase (PARG) is responsible for the catabolism of PARP-synthesized PAR to free ADP-ribose. Inhibition of PARG leads to DNA repair interruption and consequently induces cell death. This study aims to evaluate the effect of a PARG inhibitor (PARGi) on epithelial ovarian cancer (OC) cell lines, alone and in combination with a PARP inhibitor (PARPi) and/or Cisplatin.

**Methods:**

PARG mRNA levels were studied in three different OC datasets: TCGA, Hendrix, and Meyniel. PARG protein levels were assessed in 100 OC specimens from our bio-bank. The therapeutic efficacy of PARGi was assessed using cell migration and clonogenic formation assays. Flow cytometry was used to evaluate the cell apoptosis rate and the changes in the cell cycle.

**Results:**

PARG protein was highly expressed in 34% of the OC tumors and low expression was found in another 9%. Similarly, Hendrix, Meyneil and TCGA databases showed a significant up-regulation in PARG mRNA expression in OC samples as compared to normal tissue (P=0.001, P=0.005, P=0.005, respectively). The use of PARGi leads to decreased cell migration. PARGi in combination with PARPi or Cisplatin induced decreased survival of cells as compared to each drug alone. In the presence of PARPi and Cisplatin, PARG knockdown cell lines showed significant G2/M cell cycle arrest and cell death induction.

**Conclusions:**

PARG inhibition appears as a complementary strategy to PARP inhibition in the treatment of ovarian cancer, especially in the presence of homologous recombination defects.

## Introduction

Ovarian cancer (OC) is the most lethal gynecologic malignancy, with an estimated 313 959 new cases and 207 252 deaths worldwide in 2020 (1, 2). Current treatment for OC patients consists of a combination of maximal cytoreduction and platinum-taxane based chemotherapy (3). Despite these aggressive frontline treatments, the prognosis for advanced stages is poor, and the 5-year survival rate is less than 25% for women diagnosed with stages III or IV (4). Hence, new treatment strategies and paradigms are needed to deal with persistent and recurrent tumor cells, and ultimately improve prognosis.

Germline mutations in *BRCA1* or *BRCA2* genes are present in approximately 20% of patients with newly diagnosed OC (5). Recently, it has been shown that a significant proportion of sporadic tumors have a phenotype similar to the tumors found in patients with inherited *BRCA* mutations and this led to the concept of BRCAness (5). In addition to Germline mutations in *BRCA1/2* genes, BRCAness results from DNA-repair defect(s) arising from loss of homologous recombination (HR) function secondary to epigenetic perturbations such as aberrant methylation (5-31% in ovarian cancer), somatic mutations (<5%) and other abnormalities of the following HR repair genes: *TM, ATR, BARD1, BLM, BRIP1, CDK12, CHEK1, CHEK2, FANCA, FANCC, FANCD2, FANCE, FANCF, FANCI, FANCL, FANCM, MRE11, NBN, PALB2, RAD50, RAD51, RAD51B, RAD51C, RAD51D, RAD52, RAD54L*, and *RPA1* (5–8). Loss of HR function leads to impaired ability of cells to repair double-stranded DNA breaks (DSB). Inhibition of single stranded DNA repair in HR deficient cells can result in cell death by synthetic lethality (9–11). Together, HR mutations have been implicated in up to 50% of OC (5, 6, 12), representing an important therapeutic target in this disease as exemplified by the efficacy of platinum analogues, as well as the advent of PARP inhibitors, which exhibit synthetic lethality when applied to HRD cells.

Poly ADP-ribose (PAR) formation is one of the earliest events in the mechanism of DNA damage repair and is catalyzed by PARP (Poly ADP-ribose polymerase) enzymes (13–15). Additionally, PARP plays a role in cell proliferation, differentiation and transformation (16). Although the inhibition of PARP activity was initially demonstrated nearly 50 years ago, by Preiss (1971), following treatment of HeLa cells with thymidine and nicotinamide (17), the elucidation of its structure and functions had to wait for modern molecular biology techniques, which subsequently led to the screening of many potent small molecule PARP inhibitors (PARPi). While increased PARP expression and activity has been found in many different cancers, the loss of PARP activity in cells or in knockout mouse models leads to both radio and chemo-sensitisation (18, 19). PARPi trap PARP on damaged DNA site, thus interfering with the catalytic cycle of PARP, preventing DNA repair (20). Inhibition of PARP activity would lead to collapse of the replication forks and of the subsequent HR-dependent repair of these forks. Therefore, given that *BRCA1/2* mutated tumor cells have defective HR activity, the collapsed replication forks are unable to be repaired and cell death occurs (21). There are currently several PARP inhibitors approved for the treatment of *BRCA1/2* mutation carriers with ovarian, breast, prostate and pancreatic cancers (22–24). More recent studies suggest that PARPi may have much wider applications including the treatment of tumors with alternative HR deficiencies (21, 25, 26) or tumors with high levels of oxidative and replicative stress, regardless HR status (27–29). Despite the promising antitumor activity of PARPi in tumors with impaired HR repair, 40 − 70% of *BRCA1/2* mutated OC fail to respond to PARPi (10, 22, 30). Previously, we showed that PARP1 protein levels were reduced following chemotherapy *in vitro* and *in vivo* (31), which could explain in part the reported prevalent PARPi resistance (22, 30). These findings in addition to the high frequency of HR defects in OC emphasize the need to look for additional treatment options.

**Figure 1:**
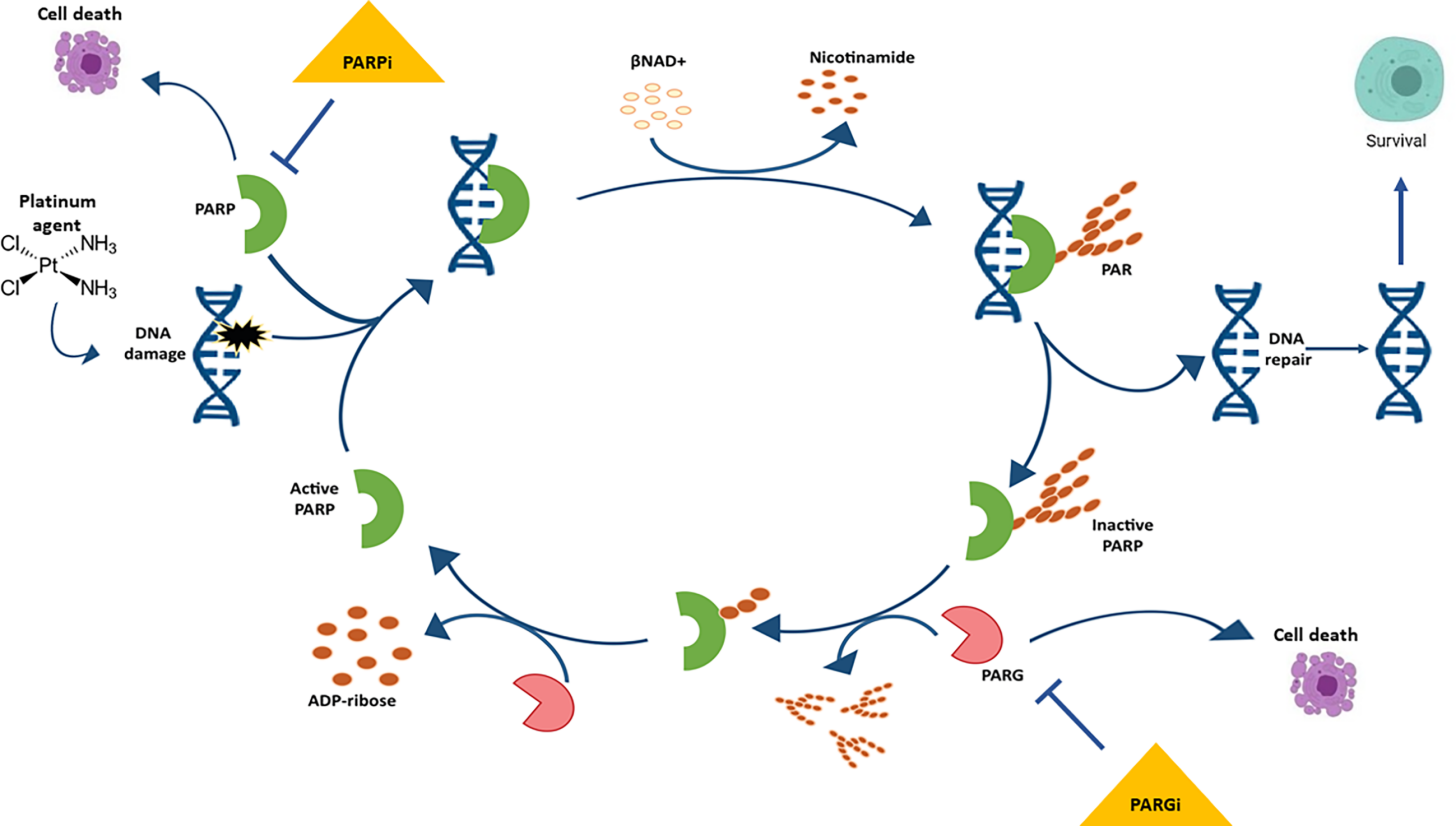
The cycle of Poly ADP-Ribose (PAR) metabolism “PARylation”. Poly ADP-ribose polymerase (PARP) binds the damaged DNA (caused by a platinum agent) and becomes active and catalyzes the formation of PAR polymers on a variety of protein acceptors, including itself. Electrostatic repulsion between the newly formed polymer and DNA causes the release of PARP, thereby inactivating it. The poly (ADP-ribose) glycohydrolase (PARG) enzyme degrades the PAR, thereby allowing for PARP to once again bind to damaged DNA and initiate “PARylation”.

Poly ADP-ribose glycohydrolase (PARG) is responsible for the catabolism of PARP-synthesized PAR to free ADP-ribose (16, 32). Like PARP and other repair proteins, PARG is recruited to sites of DNA damage and involved in the degradation of PAR by cleaving glycosidic ribose–ribose bonds within PAR chains, thus avoiding excessive PAR formation and preventing cell death (33, 34) **(** **Figure 1**). PARG deficient cells have been reported to display reduced efficiency of double strand break (DSB) and single strand break (SSB) repair, suggesting that PARG might be used as a potential target in OC (35, 36). Only a few PARG inhibitors (PARGi) are available (37) as the first selective inhibitor, PDD00017273, was developed in 2016 (38). This inhibitor was shown to have anti-tumor activity in breast, pancreatic, non-small lung cancers, and most recently in ovarian cancer (34, 36, 39–43). Moreover, we assume that unexplored synthetic lethality relationships with HRD cells may exist, and these might represent valuable drug targets for metastatic, refractory and PARPi-resistant HR-deficient tumors. By screening two pairs of *BRCA2 *isogenic cell lines with DNA repair-focused shRNA and CRISPR-based libraries, Mengwasser et al. identified APEX2 and FEN1 as synthetic lethal genes with both *BRCA1* and *BRCA2* loss-of-function (44). Another screening of the whole-genome CRISPR-Cas9 synthetic-viability/resistance was done by Dev et al. (45) in *BRCA1*-deficient breast cancer cells treated with PARP inhibitors. Two previously uncharacterized synthetic lethal proteins were identified, C20orf196 and FAM35A, whose inactivation confers strong PARP-inhibitor resistance. Most importantly, screening *in vitro* cultures derived from *BRCA2mutant* mouse mammary tumors, cell lines (KB2P1.21, KB2P3.4) and three-dimensional cancer organoids (ORG-KB2P26S.1), using DNA repair-focused shRNA and CRISPR-based libraries, confirmed PARG as a synthetic lethal gene, and loss of this gene represents a major resistance mechanism for PARPi (46).

Given the high rate of HR defects in OC, we hypothesize that inhibiting PARG may be an effective alternative therapeutic strategy for targeting specific OC cancer cells that are dependent on this activity. In addition, PARGi might increase the cytotoxicity of DNA damaging agents and may be useful against diverse ovarian malignancies, including PARPi-resistant tumors. In this study, we aimed to assess the expression of PARG in OC cells and evaluate the effect of PARGi on OC cell lines, alone and in combination with PARPi and Cisplatin.

## Methods

The study was approved by the Jewish General Hospital Research Ethics Board and all patients participating in this study gave informed consent in accordance with the JGH ethics committee regulations (protocol #15-070).

### PARG Expression

#### Gene Set Analysis (GSA)

Oncomine ™ database categorized patients according to different datasets, based on variations in gene expression patterns derived from different cDNA microarrays analysis. Ovarian cancer RNA-seq expression data were obtained from browser website (https://www.oncomine.org). Using this database, we investigated PARG mRNA levels in normal ovarian tissue and ovarian cancer cases.

#### Protein Extraction and Western Blot Analysis

In total, 100 tumor samples were analyzed including 20 ascites cell pellets, 62 primary tumors and 18 omental metastases. Snap-frozen tumor tissues were minced and lysed in lysis buffer (25mM Tris∙HCl pH7.6, 10% glycerol, 420mM NaCl, 2mM MgCl_2_, 0.5% NP-40, 0.5% Triton X-100, 1mM EDTA, protease inhibitor) on ice. Additionally, OVCAR3, SNU251, SKOV3, A2780PAR (parental), A2780CR (Cisplatin resistant), and primary tumor cell lines were harvested (2mL 0.25% Trypsin-EDTA 1x, Wisent Bio Products) and then lysed in 500μL of radio-immunoprecipitation assay (RIPA) buffer (25mM/L Tris-HCl pH 7.6, 150mM/L NaCl, 1% NP-40, 1% sodium deoxycholate, 0.1% SDS and 1mM/L EDTA). Protein concentration was determined using bicinchoninic acid assay (BCA) kit (Ref 23225, Pierce) using a spectrophotometer at 570nm.

Protein lysates (10-25μg) were separated electrophoretically on a 7.5 to 10% denaturing SDS-polyacrylamide gels and transferred to 0.2μm nitrocellulose membranes. Primary antibodies specific for *BRCA1* (Cell Signaling, Beverly, MA, USA. 1:1000), PARG (#; Cell Signaling; 1:500) and β-actin (#4967, Cell Signaling; 1:2000) were diluted in 0.1% Tween-PBS/5% Milk and put in presence of the membrane overnight at 4°C. After 3 washing (0.1%Tween-PBS1X), membranes were exposed to secondary anti-rabbit-horseradish peroxidase (HRP; L170-6515; Bio-Rad, USA; 1:10000) or anti-mouse HRP (L170-6516; Bio-Rad; 1:10000) for 1 hour at room temperature. Immunoblotting proteins were visualized using horseradish peroxidase (HRP)-conjugated secondary antibodies, and antigen-antibody complexes were detected using the Clarity™ Western ECL Substrate kit (Bio-Rad, Hercules, USA).

#### Cell Lines and Treatments

**Cell lines (** **Table 1** **):** OVCAR3 (#HTB-161), SNU-251 (#CVCL-5040) and SKOV3 (#HTB-77) were purchased from ATCC. A2780PAR and A2780CR cells were provided by Dr. Seftor (Northwestern University, Chicago).

**Table 1:** Characteristics of ovarian tumors from which cell lines were established.

Cell line	Histology	Isolated from	Treatment received	Response
OVCAR3 (47)	Serous	Ascites	CYC, CIS, DOX	Unknown
SKOV3 (48)	Adenocarcinoma	Ascites	THI	Unknown
SNU251 (49)	Endometroid	Ascites	CYC, ADR, CIS	Unknown
A2780 (50)	Unknown	Primary tumor	None	N/A

ADR, Adriamycin; CIS, Cisplatin; CYC, Cyclophosphamide; DOX, Doxurubicin; N/A, Not Applicable; THI, Thiotepa.

All the cell lines were authenticated by short tandem repeat (STR) profiling by the DNA sequencing and analysis core of the University of Colorado (51). All cell lines were frequently tested for mycoplasma infection using MycoAlert Detection Kit (Lonza #LT07-710). OVCAR3, SKOV3, A2780PAR and A2780CR display wild-type *BRCA1* genes, and SNU-251demonstrates a homozygous 1815 G>A *BRCA1* mutation (50, 52). OVCAR3 and SKOV3 were cultured in RPMI-1640 medium supplemented with 10% fetal bovine serum (FBS), 2mM glutamine, 100 U/ml penicillin, and 100μg/ml streptomycin. SNU-251 was cultured in DMEM medium supplemented with 10% FBS, 2mM glutamine, 100 U/ml penicillin, and 100μg/ml streptomycin. A2780PAR and A2780CR were cultured in RPMI-1640 medium supplemented with 10% FBS, 2mM glutamine, 1% Hepes, 100U/ml penicillin, and 100μg/ml streptomycin. A2780CR cells were maintained in media with 1μM Cisplatin every 2-3 passages to maintain Cisplatin resistance.

Patient tumor-derived ovarian cancer cells labeled GOC31 and GOC17 were isolated in our laboratory from two high-grade serous OC specimens obtained fresh at surgery. Primary cell lines were grown in OSE medium supplemented with 20% FBS and growth factors (insulin, EGFR, hydrocortisone, BPE). The cells were routinely passaged every 4 to 6 days. All cells were maintained at 37°C, in a 5% CO2, 95% air atmosphere incubator.

### Treatments

The PARGi (PDD00017273, Cat#5952) was purchased from Tocris (38). Olaparib (PARPi) (AZD2281, Cat#A10111) was purchased from AdooQ Bioscience. The drugs were diluted in DMSO (10μM and 10mM stocks respectively) and stored at -20°C. To avoid drug degradation, new aliquots were prepared directly from stocks every 5-10 uses. Cisplatin was ordered from the Jewish General Hospital Satellite Pharmacy. In a previous study (53), we showed the half maximal inhibitory concentrations (IC50) range of the same cell lines used in the current study after treatment with Olaparib, assessed by clonogenic assays. Accordingly, the final concentrations used in the present study were 0.5 and 1μM of Olaparib which is at the lower range of that used in a phase 1 clinical trial (11). For SNU251 cell line as an exception, we used a dose of 0.05 μM of Olaparib. With regard to PARGi, since there is no clinical trial reporting its plasmatic concentration, its inhibitory activity was first tested in similar range of concentrations to that employed for Olaparib. Based on our preliminary results, PARGi had a lower inhibition effect than Olaparib, and we modified the dosage accordingly, bringing the final PARGi concentrations to 0.5,1,2,5 and 10μM. Drug concentrations used for Cisplatin were 0.5μg/mL, 1μg/mL, according to the IC50 concentrations shown previously (53).

#### Generation of Stable Cell Lines

SKOV3 cells were used to generate stable cell lines with PARG knockdown. Cells were cultured to 90% confluence and transfected with lentiviral constructs expressing shRNA targeting PARG (shPARG1305, shPARG1306) (34). Twelve hours post-transfection, the cell culture medium with lentivirus were collected. SKOV3 cells were plated to 70–80% confluence and infected with lentivirus. Cells were selected with 5μg/ml puromycin for 3 days post infection. SKOV3 shPARG1305 were used for cell cycle and apoptosis assessment experiments because we observed an 80% inhibition of the PARG expression with this cell line.

#### Cell Migration Assays

Cells were grown to near confluence in 6-well adherent cell culture flat bottom plates (BD Falcon, Life Technologies). A ‘‘wound’’ was then inflicted to the cells in triplicate in each well using a sterile 200-KL pipette tip. The cells were then carefully rinsed with phosphate-buffered saline to remove any floating cells. Medium containing various concentrations of PARGi was then added. Pictures were taken of all “wounds” under an optical microscope (Olympus CKX41) at different time points (time 0, 24 and 48 hours), and the “wound” mean width was measured at three cross-sections along the length of the “wound”, using Photoshop CS3 Extended version (Adobe Systems, Inc). “Wound” closure was then calculated as a percentage value over time. At the completion of the wound healing assay, cells from the 6-well plates were collected for protein extraction and Western blotting.

#### Survival Assays

The clonogenic assay was used to determine survival fraction of cells. Briefly, 500–800 cells were plated in 6-well flat bottom cell culture plates (BD Falcon, Life Technologies). 24 hours after plating, cells were washed, and fresh medium was added in the presence or absence of increasing doses of PARGi alone and in combination with Olaparib and Cisplatin. Media containing the drug was refreshed on day 4. Colonies were fixed and stained after 7-10 days of treatment with 1.5 ml of 6% glutaraldehyde and 0.5% crystal violet and colonies were counted using the GelCount Optronix. The surviving fraction (SF) and Plating Efficiency (PE) of cells were calculated as follows (54):


$$\text{SF}=\frac{Number\;of\;colonies\;formed\;after\;treatment}{Number\;of\;cells\;seeded\;x\;Plating\;Efficiency}$$

$$\text{PE}=\frac{Number\;of\;colonies\;formed\;in\;control}{Number\;of\;cells\;seeded}$$

The interaction between PARGi, Olaparib and Cisplatin was assessed using the multiple drug effects analysis method of Chou and Talalay (55). This method quantitatively describes the interaction between two or more drugs, with combination index (CI) less than 1 indicating synergistic interactions, values greater than 1 indicating antagonistic interactions, and values equal to 1 indicating additive interactions. Calculations of the CI values were performed with CompuSyn Software (ComboSyn, Inc., Paramus, NJ. 07652 USA).

#### Cell Cycle Analysis

Cell cycle analysis was performed by propidium iodide (PI) staining for DNA content and flow cytometry analysis. For this experiment we used SKOV3-shVector and SKOV3-shPARG1305 cell lines. Briefly, 10^6^ cells were seeded in flat bottom cell culture plates (GBO, Bioscience, Frickenhausen, Germany). 24 hours after plating, fresh medium was added in the presence or absence of 2μM Olaparib or/and 1μg/mL Cisplatin. After 48 hr treatment, Hoechst 33342 was added for 30 minutes, then adherent cells were collected using trypsin-EDTA by centrifugation at 10000 rpm for 5 min and washed twice with ice cold PBS. During the last spin, 5ul PI was added for every ml of hypotonic buffer (0.1% Sodium Citrate, 0.1% Triton X-100), and incubated on ice in the dark (at least 20 min). Stained cells were analyzed (at least 20,000 events per sample) with a FACS Fortessa flow cytometer (BD BioSciences, CA). ModFit LT software (Verity Software House, Topsham, ME) was used to analyze the percentage of cells at different phases. Cells treated with DMSO (0.1%, v/v) were used as control.

#### Annexin V/PI Apoptosis Detection Assays

Apoptosis was assessed by Annexin V/PI assay using flow cytometry, according to the manufacturer’s protocol (eBioscience™ Ann exin V Apoptosis Detection Kit eFluor™ 450). Apoptotic cells were determined using the FACS Fortessa (BD BioSciences, CA) (56).

#### Statistical Analysis

Results are shown as means ± standard deviations of three independent experiments. The difference between groups was analyzed using Student’s t-test, and a p-value <0.05 was considered statistically significant.

## Results

### PARG mRNA Levels Are Over Expressed in Ovarian Cancer

**Figure 2:**
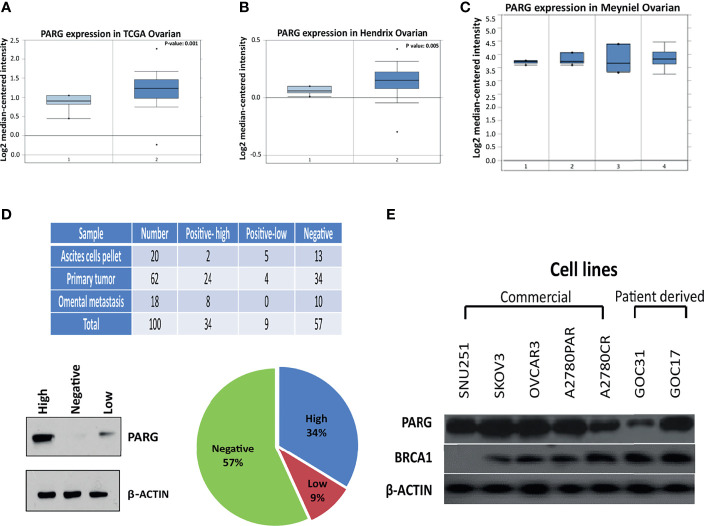
PARG is over expressed in ovarian cancer. PARG mRNA expression was evaluated in normal and malignant ovarian samples using three different datasets: the TCGA dataset (1- Normal ovary (n-8), 2 – Ovarian serous carcinoma (n-586)) **(A)** and the Hendrix dataset (1- Normal ovary (n-4), 2 – Ovarian serous carcinoma (n-41)) **(B)**, and the Meyniel dataset (1- Clear cell carcinoma (n-6), 2- Endometroid carcinoma (n-6), 3- mucinous carcinoma (n-7), 4- Serous carcinoma (n-71)) **(C)**. PARG protein levels were evaluated by western blot in 100 high grade serous ovarian cancer tumors kept in our biobank. PARG representative western blot for each level category (low, high, and negative). The level category was set according to the intensity of the western blot band while OVCAR3 protein extract was used as a positive control. **(D)**. Expression of PARG and BRCA1 proteins were examined by western blot **(E)** in commercial (SNU251, SKOV3, OVCAR3, A2780PAR, A2780CR) and patients derived (GOC17, GOC31) cell lines.

We initially evaluated the PARG mRNA expression in normal ovarian samples compared to high grade serous adenocarcinoma samples using two different datasets of ONCOMINE database: the TCGA dataset (586 cases) (**Figure 2A**) and Hendrix dataset (41 cases) (**Figure 2B**). Both datasets showed a significant over expression of PARG mRNA in the malignant cases (P=0.001, P=0.005 respectively). Next, we evaluated the expression of PARG mRNA in different histological subtypes using Meyniel dataset and found relatively higher expression levels of PARG mRNA in serous adenocarcinoma cases compared to other histological subtypes like endometrioid, mucinous, and clear cell adenocarcinoma (**Figure 2C**).

### PARG Is Expressed at the Protein Level in Commercial and Tumor-Derived Ovarian Cell Lines

We evaluated PARG protein levels in 100 unselected snap-frozen high grade OC tumors kept in our biobank and checked the level of PARG in commercial (OVCAR3, SNU251, SKOV3, A2780PAR and A2780CR) and primary cell lines derived from patient tumors (GOC31 and GOC17). Baseline characteristics of the study population are displayed in **Table 2**.

**Table 2:** Baseline characteristics of the cohort.

	Total (n-100)	Negative (n-57)	Positive low (n-9)	Positive high (n-34)	P-Value
**Age, mean (SD)**	59.6 ± 13.6	59.9 ± 13.0	61.3 ± 14.2	58.8 ± 14.7	0.8
**BMI, mean (SD)**	28.8 ± 6.0	28.8 ± 5.5	28.9 ± 8.8	28.8 ± 6.3	1.0
**Stage:** **Early (I/II)** **Advanced (III/IV)**	27 (27.0%)	17 (63.0%)	2 (7.4%)	8 (29.6%)	0.7
	73 (73.0%)	40 (54.8%)	7 (9.6%)	26 (35.6%)	
**CA125, mean (SD)**	1340.1 ± 2260.2	1237.4 ± 2032.2	1566.5 ± 1501.5	1452.5 ± 2779.4	0.8
**Histology**					0.4
**Serous**	74 (74.0%)	41 (55.4%)	4 (5.4%)	29 (39.2%)	
**Clear cell**	17 (17.0%)	11 (19.3%)	2 (11.8%)	4 (23.5%)	
**Endometroid**	9 (9.0%)	5 (55.6%)	3 (33.3%)	1 (11.1%)	
**Debulking:** Optimal* **Non-optimal**	92 (92.0%)	53 (57.6%)	8 (8.7%)	31 (33.7%)	0.9
	8 (8.0%)	4 (50.0%)	1 (11.1%)	3 (37.5%)	
**Platinum sensitivity** Sensitive** **Resistant/refractory**	74 (74.0%)	45 (60.8%)	5 (6.8%)	24 (32.4%)	0.2
	26 (26.0%)	12 (46.2%)	4 (15.4%)	10 (38.5%)	

BMI, body mass index. *Optimal debulking- residual disease < 1mm. ** platinum sensitive- cancer that responds to platinum-based treatment and if it comes back, it come 6 or more months after treatment.

Western blot analysis showed high expression level of PARG protein in 34% and low expression in 9% of the tumors (**Figure 2D**). In standard culture conditions, ovarian commercial and tumor-derived cell lines showed different expression levels of PARG protein (**Figure 2E**) and noticeably, the *BRCA1* protein was at a very low level in SNU251 cells.

### Inhibition of PARG Impairs Ovarian Cancer Cell Migration

**Figure 3:**
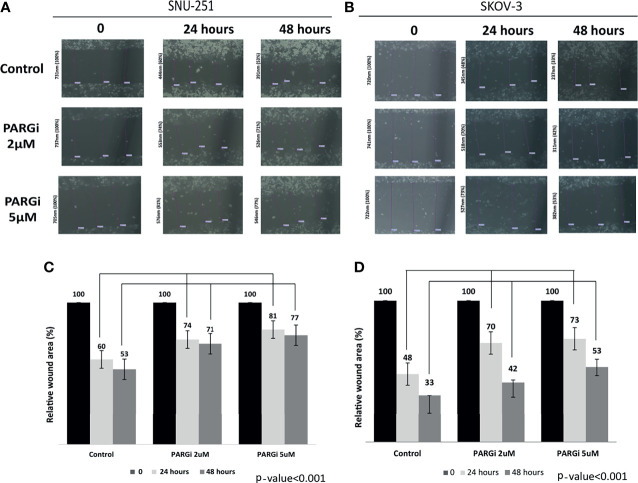
Effect of PARG inhibitor on cell migration. ‘‘Wounds’’ were made on monolayers of SNU251 **(A)** and SKOV3 **(B)** cells grown to near confluence. Cells were then incubated in their media containing PARG inhibitor 2 and 5μM for 24 and 48 hours. Treated or untreated (control) cells were photographed only after scratch (time 0) and after 24 and 48 hours. Results presented here are representative of triplicate independent samples of each cell line. The rate of migration was measured by quantifying the total distance that the cells (as indicated by rulers) moved from the edge of the scratch toward the center of the scratch. A value of 100% was given to the wound area at time 0. The migration of treated samples was compared with wound area at time 0. Bar graph recapitulating the percent of “Wound” closure that was calculated over time. P values were calculated by two-tailed t-test **(C, D)**.

Wound-healing assays were performed to investigate the potential inhibitory effect of PARGi on cell migration of *BRCA* proficient (SKOV3) and *BRCA* deficient (SNU251) cell lines. Results indicate that the migration of both cell types was inhibited by PARGi reaching a maximum at 48 hours, at which time the ‘‘wound’’ of SKOV3 cells remained 42% (2μM) and 53% (5μM) open as compared with 33% in untreated cells at the same time, suggesting slower cell mobility (**Figures 3A, C**) (p-value<0.001). More prominent results were found with SNU251 cells: 71% (2μM) and 77% (μM) wound opening in the presence of PARGi versus 53% in the untreated controls (**Figures 3B, D**), (p-value<0.001). These results indicate that PARGi slows the migration of these 2 cell lines in a time-dependent manner.

### PARGi Decreases Survival of OC Cells When Combined With Olaparib and Cisplatin

**Figure 4:**
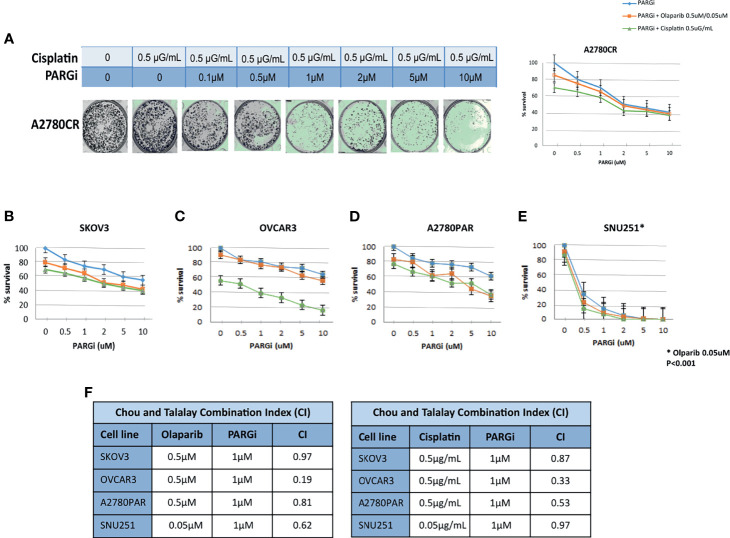
PARG inhibitor (PARGi) sensitizes ovarian cancer cells to Olaparib and Cisplatin. Survival curves: Blue- increasing doses of PARGi 0, 0.5, 1, 2, 5, and 10 μM. Orange- increasing doses of PARGi 0, 0.5, 1, 2, 5, and 10 μM + Olaparib 0.5 μM (0.05 μM for SNU251). Green- increasing doses of PARGi 0, 0.5, 1, 2, 5, and 10 μM + Cisplatin 0.5 μg/mL In a clonogenic assay at day 7-10, the sensitivity to combination treatments including(PARGi+Olaparib) and (PARGi+Cisplatin) is higher compared to PARGi, Olaparib and Cisplatin alone. PARGi re-synthesize A2780CR to Cisplatin **(A)**. BRCA mutated cells “SNU251” **(E)** were more sensitive compared to BRCA wild-type cells “SKOV3” “OVCAR3” “A2780PAR” **(B–D)**. The evaluation of combination index (CI) for PARGi, Olaparib and Cisplatin **(F)** was calculated where CI<1 indicates synergy between the drugs and CI>1 indicates an additive effect. Results are presented as means ± SEM for triplicates of three independent experiments.*p < 0.001, Olaparib dose 0.05uM.

We next evaluated the sensitivity of the OC cells to PARGi, alone and in combination with Olaparib and Cisplatin by clonogenic assays. All cell lines we used (SKOV3, OVCAR3, SNU-251, A2780PAR and A2780CR) were treated with increasing doses of PARGi (0.1–10μM), alone and in combination with olaparib (0.5μM) or cisplatin (0.5μg/mL). Decreased survival of OC cells was shown with combination treatment (PARGi+Olaparib/PARGi+Cisplatin) as compared to single treatments (**Figures 4B–E**). A2780CR cells are well known to be resistant to platinum agents. Interestingly, treatment with PARGi re-sensitize these cells to Cisplatin, as shown in **Figure 4A**. Furthermore, the percent of survival values of *BRCA* mutated SNU-251 cells in each experiment was greatly diminished compared with that of other cell lines (**Figures 4A–E**).

To further determine the nature of the interaction between PARGi, Olaparib and Cisplatin, we used the multiple drug effects analysis method of Chou and Talalay (55). In all cell lines tested, we calculated a combination index (CI) between (0.19-0.97), with any number <1 indicating a synergistic effect (**Figure 4F**).

### PARG Silencing Induces G2/M Arrest and Cell Death in Ovarian Cancer Cells Treated With Olaparib and Cisplatin

**Figure 5:**
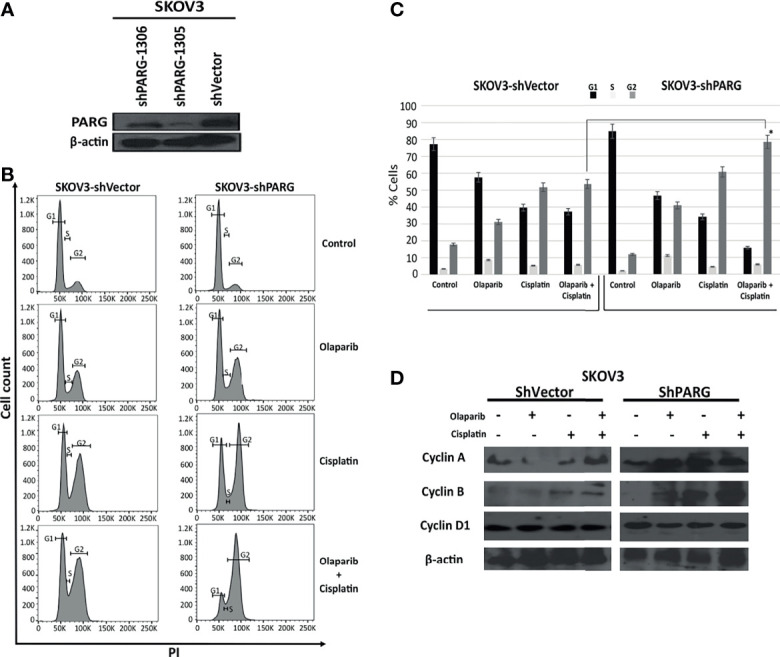
PARG silencing induces G2/M arrest in ovarian cancer cells treated with Olaparib and Cisplatin. Expression of PARG in SKOV3-shVector and SKOV3_shPARG cell lines **(A)**. SKOV3-shVector and SKOV3_shPARG cells were treated with Olaparib 2 μM, Cisplatin 1μg/mL and combination of Olaparib and Cisplatin for 48 hours. Cells were synchronized, and cell cycle analysis were performed using flow cytometry **(B, C)**. Protein expression of cell cycle related proteins (cyclin A, cyclin B and cyclin D1) were examined by western blot **(D)**. Results are presented as means ± SEM for triplicates of three independent experiments, *p value < 0.05.

To further decipher the mechanism of the anti-tumorigenic activity of PARGi, we evaluated the effect of PARG inhibition on the regulation of apoptosis and cell cycle. Olaparib 2 μM/Cisplatin 1μg/mL combination treatment resulted in G2/M arrest in up to 78.4% in the SKOV3-shPARG1305 and 53.5% in SKVO3-shVector compared to 40.8%, 31.2% with Olaparib alone and 60.7%, 51.7% with Cisplatin alone and 11.7%, 17.8% without treatment, respectively, (P<0.001) (**Figures 5A–C**). The G2/M arrest was also confirmed at protein level by evaluating cyclins A, B, D1 with western blot (**Figure 5D**). Cyclin D1 is a protein required for cell cycle G1/S transition. Cyclin A resides in the nucleus during S phase where it is involved in the initiation and completion of DNA replication. Cyclin A remains associated with CDK1 from late S into late G2 phase when it is replaced by cyclin B. Cyclin B is a mitotic cyclin and is necessary for the progression of the cells into and out of M phase. While a stable level of cyclin D1 expression was observed, an increase in cyclin A and cyclin B was induced after Olaparib/Cisplatin treatment in SKOV3-ShPARG1305 cells as compared with ShVector control treated with the same regimen.

**Figure 6:**
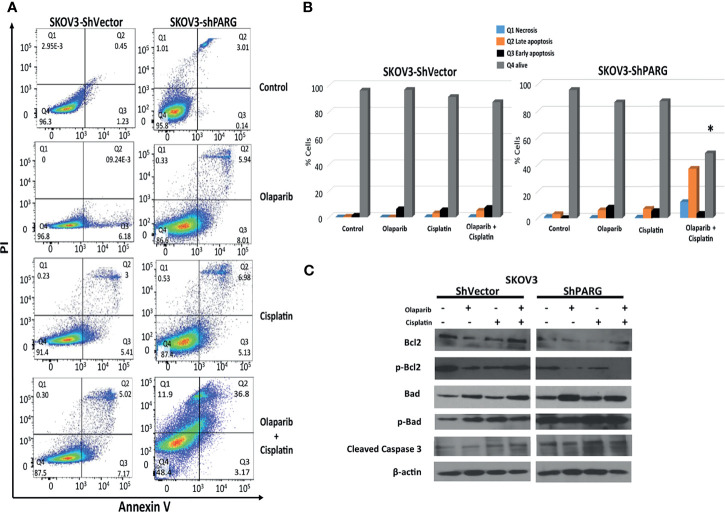
PARG silencing induces apoptosis in ovarian cancer cells treated with Olaparib and Cisplatin. SKOV3-shVector and SKOV3-shPARG cells were treated with Olaparib 2 μM, Cisplatin 1μg/mL and combination of Olaparib and Cisplatin for 48 hours, then apoptotic rates were assessed using Annexin V/PI double staining followed by flow cytometry analysis **(A, B)**. Protein expression of pro and anti-apoptotic proteins (Bcl2, p-Bcl, Bad, p-Bad, cleaved caspase3) were examined by western blot **(C)**. Results are presented as means ± SEM for triplicates of three independent experiments, *p value < 0.05.

We further investigated the effect of the treatments in modulating apoptosis. First, we studied its effects by quantifying the apoptotic cells using Annexin V/PI double staining assay (**Figure 6A**). We found Olaparib monotherapy induced cell death in ~14%, Cisplatin in ~12% and the combination of Olaparib/Cisplatin in ~53% of SKOV3-ShPARG1305 cells compared to ~%, ~9% and ~13% in the SKOV3-ShVector, respectively (**Figure 6B**). We also evaluated the pro-survival proteins Bcl2 and p-Bcl2, and our results showed significant down regulation of these proteins in SKOV3-ShPARG1305 cells, while pro-apoptotic proteins Bad, p-Bad, and cleaved caspase-3 were up regulated, all after treatment with Olaparib and Cisplatin alone and in combination (**Figure 6C**). These results suggest that increased PARG inhibition correlated with cell cycle arrest and induction of apoptosis.

## Discussion

The cell lines used in this study represented both *BRCA* deficient (SNU251) and wild type *BRCA* proficient (SKOV3, OVCAR3, A2780PAR and A2780CR) ovarian cancers. Results of this study suggest that PARGi reduces cell migration and suppresses formation of clones in *BRCA* proficient and deficient ovarian cell lines. In addition, knocking down PARG promotes G2/M arrest and cell death when cells are exposed to PARPi as well as DNA damaging agents (Cisplatin).

In order to spread and disseminate throughout the body, ovarian cancer cells must migrate and invade through extracellular matrix, intravasate into blood circulation, attach to a distant site, and finally extravasate to form distant foci; cell migration is a key property for the development of this process. In this study, we observed the inhibitory effect of PARGi on cell migration in a time- and concentration-dependent manner, in support of our conclusion that the inhibitory effect of PARGi on cell migration is genuine and is not only due to cell death.

The mechanisms by which PARG inhibition affects cancer cells remain elusive with various reported potential mechanisms; (1) HeLa-derived PARG deficient cells exhibited enhanced sensitivity to radiotherapy, caused by defects in the repair of single and double-strand breaks and in mitotic spindle checkpoint, leading to alteration of progression of mitosis (57); (2) PARG deficiency sensitized mouse embryonic stem cells to linear-energy-transfer radiation through the defective repair of double-strand breaks resulted in the induction of apoptosis (58); (3) PARG inhibition in the CF7 breast adenocarcinoma cell line increases endogenous DNA damage, stalls replication forks and increases homologous recombination. The authors proposed that it is the lack of HR proteins at the PARGi-induced stalled replication forks that induces cell death (39). Recently it was hypothesized that sensitivity of OC cells arises due to an underlying DNA replication vulnerability that renders cells dependent on PARG activity, such that upon PARG inhibition, stalled DNA replication forks fail to restart, leading to persistent replication stress and DNA damage (43).

All five commercial cell lines we investigated in our study are widely used in ovarian cancer research. However, only OVCAR3 is undoubtedly of high grade serous ovarian cancer origin. Although SKOV3 line is frequently cited as “serous’’, it has been only vaguely reported in the original paper as “adenocarcinoma cell line derived from the ascitic fluid of ovarian cancer patient” (59). In addition, the A2780 was originally described as a cell line established from an “ovarian endometrioid adenocarcinoma tumor” (60). The histologic diversity of the included cell lines can provide an explanation for the different responses to treatments used in this study and it can also explain the significant lower CI of OVCAR3 that is in keeping with high grade serous ovarian cancer which is a highly PARPi and platinum sensitive tumor. The effect of PARGi on cancer cell cycle remains unclear as well. Nakadate et al. demonstrated that depletion of PARG led to the abrogation of radiation-induced G2/M arrest and checkpoint activation in lung and prostate cancers cells (41). G2/M arrest is induced transiently to protect cells from DNA damage. The abrogation of the G2/M checkpoint leads to a decrease in DNA repair resulting in cell death (61). Ame et al. reported that HeLa cells treated with radiotherapy had an increased G2/M arrest and accumulation of cells in metaphase (57). Consistent with Ame’s et al. findings, in the present study we found that PARG silencing led to induction of G2/M arrest in the presence of PARPi and DNA damaging agents, resulting in accumulation of PAR, a delay in the repair of DNA strand breaks and mitotic defects, generating polyploid cells or causing cell death by mitotic catastrophe.

PARPi introduction has made considerable progress in the clinical outcomes of ovarian cancer. The recognition that certain molecular pathways including the PAR metabolism are critical to carcinogenesis has triggered a revolution in ovarian cancer drug development. However, PARPi resistance continues to be a significant challenge and it is well-recognized that the failure of PARPi arises due to an inability to induce apoptosis at a cellular level. In this study, it has been shown that different OC cell lines responds better when PARG is silenced, suggesting that PARGi can maximize the benefit of chemotherapy and delay the process of chemo-resistance. By knocking down PARG, the ratio of pro-apoptotic Bcl-2 family members (Bax, Bad) was favored to anti-apoptotic Bcl-2 family (Bcl-2 and Bcl-XL) members, with increased cell apoptosis as indicated by flow cytometry analysis and western blot.

Our observations validate the potential anti-tumor role of PARGi in the treatment of ovarian cancer which was shown recently by Pillay et al. (43). By employing apoptosis, cell cycle and clonogenic assays, on a subset of OC cell lines, Pillay confirmed the synthetic lethality of PARGi with inhibition of DNA replication factors and inducing cell death. The key question is whether PARG inhibitors will offer dissimilar therapeutic opportunities compared with PARP inhibitors in the treatment of cancer. Pillay et al. (43), Gogola et al. (46) and Gravelles et al. (39) showed that these two modalities are differentiated, with several ovarian and breast cancers cell lines sensitive to one but not the other. Interestingly, Gogola et al. showed that loss of (PARG) induces PARPi resistance in *BRCA2*-mutated mouse mammary tumors by restoring PARP1 signaling. Be at variance with these findings, in the current study we observed a synergistic interaction between PARGi and PARPi in all cell lines. This dissimilar interaction might be explained by the difference in cell lines and drug doses used in the studies: while we chose SKOV3, A2780PAR, A2780CR and OVCAR3 cell lines to represent *BRCA* wild type serous ovarian cancer, and SNU251 which is an endometroid ovarian cancer cell line that was previously reported to carry a nonsense mutation at amino acid 1815 of *BRCA1*. Pillay et al. assembled a panel of six serous ovarian cell lines, 3 are reported to have *BRCA1/2* mutation: Kuramochi (*BRCA2mutant*), OVSAHO (*BRCA2mutant*), COV362 (*BRCA1mutant*) and 3 *BRCA* wild type cell lines: COV318, CAOV3, and OVCAR3. Lastly, Gogola’s group used two types of *in vitro* cultures that they derived from BRCA2 -/-; Trp53-/- mouse mammary tumors from K14cre;Trp53F/F;BRCA2F/F (KB2P) mice: two-dimensional (2D) tumor cell lines (KB2P1.21,KB2P3.4) and three-dimensional (3D) cancer organoids (ORG-KB2P26S.1). In regard to treatment protocol, while “PDD00017273” was used in all studies, the doses used in our study were remarkably different from the others. Unlike Gogola and Pillay who used one fixed dose of 1µM in all experiments, we used a wider range of doses, and we showed an increased effect of the treatment in the higher doses (2, 5 and 10 µM).

Another added value of our study include: 1- this study opens a window to the potential clinical benefit of PARGi as we report on high expression of PARG in ovarian cancer cells using novel analysis of online databases and in patient derived samples. 2-our results show that PARGi also inhibits cancer cells migration in addition to capability to induce cell death.

Limitations include: the use of commercial cell lines can differ from real patient’s tumors which are often more heterogeneous. We used *BRCA*1 deficient cell-line (SNU-251), however in further studies it will be interesting to evaluate the influence of *BRCA2* mutation and evaluate xenograft models.

## Conclusions

This study shows that in ovarian cancer, PARG inhibition reduces cell migration, suppresses clone formation, and promotes G2/M cell cycle arrest and cell death, alone and in combination with PARPi and Cisplatin. PARG inhibitors suitable for clinical evaluation are not yet available. Our results, however, support the potential use of PARG inhibitors as viable, complementary strategy to induce cell lethality and invasion arrest in ovarian cancer and potentially other HR-deficient cancers.

## Data Availability Statement

The raw data supporting the conclusions of this article will be made available by the authors, without undue reservation.

## Ethics Statement

The studies involving human participants were reviewed and approved by The Medical/Biomedical Research Ethics and Committee of the CIUSSS West-Central Montreal research ethics board at the Jewish General Hospital, Montreal, Canada. The patients/participants provided their written informed consent to participate in this study.

## Author Contributions

EM, TB, LK, RK, and FR have performed clonogenic and proliferation assay. EM, AY, OR, and LK did transfection studies and analyzed the data. VL-O carried out in silica analysis and contribute to the analysis of the results. EM, TB, and AK have carried out apoptosis and cell cycle experiments. EM and VL-O wrote the manuscript and analyzed the results. WG, SL, and SS obtained all the clinical samples and revised and edited the manuscript. AY and WG designed and supervised the study and helped drafting the manuscript. All authors made substantial contributions to the conception and design, data acquisition, data analysis and revision of the intellectual content of the manuscript. In addition, each author has agreed to be accountable for the accuracy and integrity of this research work. All authors contributed to the article and approved the submitted version.

## Funding

This work was made possible in part by grants from the Montreal-Israel Cancer Research Foundation, Gloria’s Girls and the Susan and Jonathan Fund. The funding bodies had no role in the design of the study, collection, analysis, interpretation of data, or in writing the manuscript.

## Conflict of Interest

The authors declare that the research was conducted in the absence of any commercial or financial relationships that could be construed as a potential conflict of interest.

## Publisher’s Note

All claims expressed in this article are solely those of the authors and do not necessarily represent those of their affiliated organizations, or those of the publisher, the editors and the reviewers. Any product that may be evaluated in this article, or claim that may be made by its manufacturer, is not guaranteed or endorsed by the publisher.

## References

[1] (2020). Available at: https://gco.iarc.fr/today/data/factsheets/populations/900-world-fact-sheets.pdf.

[2] Siegel RL Miller KD Jemal A. Cancer Statistics, 2020. CA Cancer J Clin (2020) 70(1):7–30. doi: 10.3322/caac.21590 31912902

[3] Wilson MK Pujade-Lauraine E Aoki D Mirza MR Lorusso D Oza AM. Fifth Ovarian Cancer Consensus Conference of the Gynecologic Cancer InterGroup: Recurrent Disease. Ann Oncol (2017) 28(4):727–32. doi: 10.1093/annonc/mdw663 PMC6246494 27993805

[4] Torre LA Bray F Siegel RL Ferlay J Lortet-Tieulent J Jemal A. Global Cancer Statistics, 2012. CA Cancer J Clin (2015) 65(2):87–108. doi: 10.3322/caac.21262 25651787

[5] Hennessy BT Timms KM Carey MS Gutin A Meyer LA Flake DD 2nd. Somatic Mutations in BRCA1 and BRCA2 Could Expand the Number of Patients That Benefit From Poly (ADP Ribose) Polymerase Inhibitors in Ovarian Cancer. J Clin Oncol (2010) 28(22):3570–6. doi: 10.1200/JCO.2009.27.2997 PMC2917312 20606085

[6] Cancer Genome Atlas Research Network. Integrated Genomic Analyses of Ovarian Carcinoma. Nature (2011) 474(7353):609–15. doi: 10.1038/nature10166 PMC3163504 21720365

[7] Coleman RL Oza AM Lorusso D Aghajanian C Oaknin A Dean A. Rucaparib Maintenance Treatment for Recurrent Ovarian Carcinoma After Response to Platinum Therapy (ARIEL3): A Randomised, Double-Blind, Placebo-Controlled, Phase 3 Trial. Lancet (2017) 390(10106):1949–61. doi: 10.1016/S0140-6736(17)32440-6 PMC5901715 28916367

[8] Bajrami I Frankum JR Konde A Miller RE Rehman FL Brough R. Genome-Wide Profiling of Genetic Synthetic Lethality Identifies CDK12 as a Novel Determinant of PARP1/2 Inhibitor Sensitivity. Cancer Res (2014) 74(1):287–97. doi: 10.1158/0008-5472.CAN-13-2541 PMC4886090 24240700

[9] Fong PC Boss DS Yap TA Tutt A Wu P Mergui-Roelvink M. Inhibition of Poly(ADP-Ribose) Polymerase in Tumors From BRCA Mutation Carriers. N Engl J Med (2009) 361(2):123–34. doi: 10.1056/NEJMoa0900212 19553641

[10] Audeh MW Carmichael J Penson RT Friedlander M Powell B Bell-McGuinn KM. Oral Poly(ADP-Ribose) Polymerase Inhibitor Olaparib in Patients With BRCA1 or BRCA2 Mutations and Recurrent Ovarian Cancer: A Proof-of-Concept Trial. Lancet (2010) 376(9737):245–51. doi: 10.1016/S0140-6736(10)60893-8 20609468

[11] Fong PC Yap TA Boss DS Carden CP Mergui-Roelvink M Gourley C. Poly(ADP)-Ribose Polymerase Inhibition: Frequent Durable Responses in BRCA Carrier Ovarian Cancer Correlating With Platinum-Free Interval. J Clin Oncol (2010) 28(15):2512–9. doi: 10.1200/JCO.2009.26.9589 20406929

[12] Press JZ De Luca A Boyd N Young S Troussard A Ridge Y. Ovarian Carcinomas With Genetic and Epigenetic BRCA1 Loss Have Distinct Molecular Abnormalities. BMC Cancer (2008) 8:17. doi: 10.1186/1471-2407-8-17 18208621 PMC2245962

[13] Dziadkowiec KN Gasiorowska E Nowak-Markwitz E Jankowska A. PARP Inhibitors: Review of Mechanisms of Action and BRCA1/2 Mutation Targeting. Prz Menopauzalny (2016) 15(4):215–9. doi: 10.5114/pm.2016.65667 PMC5327624 28250726

[14] Luo X Kraus WL. On PAR With PARP: Cellular Stress Signaling Through Poly(ADP-Ribose) and PARP-1. Genes Dev (2012) 26(5):417–32. doi: 10.1101/gad.183509.111 PMC3305980 22391446

[15] Robert I Karicheva O Reina San Martin B Schreiber V Dantzer F. Functional Aspects of PARylation in Induced and Programmed DNA Repair Processes: Preserving Genome Integrity and Modulating Physiological Events. Mol Aspects Med (2013) 34(6):1138–52. doi: 10.1016/j.mam.2013.02.001 23454615

[16] D’Amours D Desnoyers S D’Silva I Poirier GG. Poly(ADP-Ribosyl)Ation Reactions in the Regulation of Nuclear Functions. Biochem J (1999) 342(Pt 2):249–68. PMC1220459 10455009

[17] Preiss J Schlaeger R Hilz H. Specific Inhibition of Poly Adpribose Polymerase by Thymidine and Nicotinamide in HeLa Cells. FEBS Lett (1971) 19(3):244–6. doi: 10.1016/0014-5793(71)80524-0 11946222

[18] Miknyoczki SJ Jones-Bolin S Pritchard S Hunter K Zhao H Wan W. Chemopotentiation of Temozolomide, Irinotecan, and Cisplatin Activity by CEP-6800, a Poly(ADP-Ribose) Polymerase Inhibitor. Mol Cancer Ther (2003) 2(4):371–82. 12700281

[19] de Murcia JM Niedergang C Trucco C Ricoul M Dutrillaux B Mark M. Requirement of Poly(ADP-Ribose) Polymerase in Recovery From DNA Damage in Mice and in Cells. Proc Natl Acad Sci USA (1997) 94(14):7303–7. doi: 10.1073/pnas.94.14.7303 PMC23816 9207086

[20] Murai J Huang SY Das BB Renaud A Zhang Y Doroshow JH. Trapping of PARP1 and PARP2 by Clinical PARP Inhibitors. Cancer Res (2012) 72(21):5588–99. doi: 10.1158/0008-5472.CAN-12-2753 PMC3528345 23118055

[21] Bryant HE Schultz N Thomas HD Parker KM Flower D Lopez E. Specific Killing of BRCA2-Deficient Tumours With Inhibitors of Poly(ADP-Ribose) Polymerase. Nature (2005) 434(7035):913–7. doi: 10.1038/nature03443 15829966

[22] Ledermann J Harter P Gourley C Friedlander M Vergote I Rustin G. Olaparib Maintenance Therapy in Platinum-Sensitive Relapsed Ovarian Cancer. N Engl J Med (2012) 366(15):1382–92. doi: 10.1056/NEJMoa1105535 22452356

[23] Loibl S O’Shaughnessy J Untch M Sikov WM Rugo HS McKee MD. Addition of the PARP Inhibitor Veliparib Plus Carboplatin or Carboplatin Alone to Standard Neoadjuvant Chemotherapy in Triple-Negative Breast Cancer (BrighTNess): A Randomised, Phase 3 Trial. Lancet Oncol (2018) 19(4):497–509. doi: 10.1016/S1470-2045(18)30111-6 29501363

[24] Sandhu SK Omlin A Hylands L Miranda S Barber LJ Riisnaes R. Poly (ADP-Ribose) Polymerase (PARP) Inhibitors for the Treatment of Advanced Germline BRCA2 Mutant Prostate Cancer. Ann Oncol (2013) 24(5):1416–8. doi: 10.1093/annonc/mdt074 23524863

[25] Jonsson P Bandlamudi C Cheng ML Srinivasan P Chavan SS Friedman ND. Tumour Lineage Shapes BRCA-Mediated Phenotypes. Nature (2019) 571(7766):576–9. doi: 10.1038/s41586-019-1382-1 PMC7048239 31292550

[26] Turner NC Lord CJ Iorns E Brough R Swift S Elliott R. A Synthetic Lethal siRNA Screen Identifying Genes Mediating Sensitivity to a PARP Inhibitor. EMBO J (2008) 27(9):1368–77. doi: 10.1038/emboj.2008.61 PMC2374839 18388863

[27] Michelena J Lezaja A Teloni F Schmid T Imhof R Altmeyer M. Analysis of PARP Inhibitor Toxicity by Multidimensional Fluorescence Microscopy Reveals Mechanisms of Sensitivity and Resistance. Nat Commun (2018) 9(1):2678. doi: 10.1038/s41467-018-05031-9 29992957 PMC6041334

[28] Majuelos-Melguizo J Rodriguez MI Lopez-Jimenez L Rodriguez-Vargas JM Marti Martin-Consuegra JM Serrano-Saenz S. PARP Targeting Counteracts Gliomagenesis Through Induction of Mitotic Catastrophe and Aggravation of Deficiency in Homologous Recombination in PTEN-Mutant Glioma. Oncotarget (2015) 6(7):4790–803. doi: 10.18632/oncotarget.2993 PMC4467115 25576921

[29] Schoonen PM Talens F Stok C Gogola E Heijink AM Bouwman P. Progression Through Mitosis Promotes PARP Inhibitor-Induced Cytotoxicity in Homologous Recombination-Deficient Cancer Cells. Nat Commun (2017) 8:15981. doi: 10.1038/ncomms15981 28714471 PMC5520019

[30] Gelmon KA Tischkowitz M Mackay H Swenerton K Robidoux A Tonkin K. Olaparib in Patients With Recurrent High-Grade Serous or Poorly Differentiated Ovarian Carcinoma or Triple-Negative Breast Cancer: A Phase 2, Multicentre, Open-Label, non-Randomised Study. Lancet Oncol (2011) 12(9):852–61. doi: 10.1016/S1470-2045(11)70214-5 21862407

[31] Marques M Beauchamp MC Fleury H Laskov I Qiang S Pelmus M. Chemotherapy Reduces PARP1 in Cancers of the Ovary: Implications for Future Clinical Trials Involving PARP Inhibitors. BMC Med (2015) 13:217. doi: 10.1186/s12916-015-0454-9 26354718 PMC4565010

[32] Brochu G Duchaine C Thibeault L Lagueux J Shah GM Poirier GG. Mode of Action of Poly(ADP-Ribose) Glycohydrolase. Biochim Biophys Acta (1994) 1219(2):342–50. doi: 10.1016/0167-4781(94)90058-2 7918631

[33] Tallis M Morra R Barkauskaite E Ahel I. Poly(ADP-Ribosyl)Ation in Regulation of Chromatin Structure and the DNA Damage Response. Chromosoma (2014) 123(1-2):79–90. doi: 10.1007/s00412-013-0442-9 24162931

[34] Marques M Jangal M Wang LC Kazanets A da Silva SD Zhao T. Oncogenic Activity of Poly (ADP-Ribose) Glycohydrolase. Oncogene (2019) 38(12):2177–91. doi: 10.1038/s41388-018-0568-6 PMC6484711 30459355

[35] Erdelyi K Bai P Kovacs I Szabo E Mocsar G Kakuk A. Dual Role of Poly(ADP-Ribose) Glycohydrolase in the Regulation of Cell Death in Oxidatively Stressed A549 Cells. FASEB J (2009) 23(10):3553–63. doi: 10.1096/fj.09-133264 PMC2747681 19571039

[36] Fathers C Drayton RM Solovieva S Bryant HE. Inhibition of Poly(ADP-Ribose) Glycohydrolase (PARG) Specifically Kills BRCA2-Deficient Tumor Cells. Cell Cycle (2012) 11(5):990–7. doi: 10.4161/cc.11.5.19482 22333589

[37] Genovese T Di Paola R Catalano P Li JH Xu W Massuda E. Treatment With a Novel Poly(ADP-Ribose) Glycohydrolase Inhibitor Reduces Development of Septic Shock-Like Syndrome Induced by Zymosan in Mice. Crit Care Med (2004) 32(6):1365–74. doi: 10.1097/01.CCM.0000127775.70867.0C 15187521

[38] James DI Smith KM Jordan AM Fairweather EE Griffiths LA Hamilton NS. First-In-Class Chemical Probes Against Poly(ADP-Ribose) Glycohydrolase (PARG) Inhibit DNA Repair With Differential Pharmacology to Olaparib. ACS Chem Biol (2016) 11(11):3179–90. doi: 10.1021/acschembio.6b00609 27689388

[39] Gravells P Grant E Smith KM James DI Bryant HE. Specific Killing of DNA Damage-Response Deficient Cells With Inhibitors of Poly(ADP-Ribose) Glycohydrolase. DNA Repair (Amst) (2017) 52:81–91. doi: 10.1016/j.dnarep.2017.02.010 28254358 PMC5360195

[40] Gravells P Neale J Grant E Nathubhai A Smith KM James DI. Radiosensitization With an Inhibitor of Poly(ADP-Ribose) Glycohydrolase: A Comparison With the PARP1/2/3 Inhibitor Olaparib. DNA Repair (Amst) (2018) 61:25–36. doi: 10.1016/j.dnarep.2017.11.004 29179156 PMC5765821

[41] Nakadate Y Kodera Y Kitamura Y Tachibana T Tamura T Koizumi F. Silencing of Poly(ADP-Ribose) Glycohydrolase Sensitizes Lung Cancer Cells to Radiation Through the Abrogation of DNA Damage Checkpoint. Biochem Biophys Res Commun (2013) 441(4):793–8. doi: 10.1016/j.bbrc.2013.10.134 24211580

[42] Jain A Agostini LC McCarthy GA Chand SN Ramirez A Nevler A. Poly (ADP) Ribose Glycohydrolase Can Be Effectively Targeted in Pancreatic Cancer. Cancer Res (2019) 79(17):4491–502. doi: 10.1158/0008-5472.CAN-18-3645 PMC6816506 31273064

[43] Pillay N Tighe A Nelson L Littler S Coulson-Gilmer C Bah N. DNA Replication Vulnerabilities Render Ovarian Cancer Cells Sensitive to Poly(ADP-Ribose) Glycohydrolase Inhibitors. Cancer Cell (2019) 35(3):519–33.e8. doi: 10.1016/j.ccell.2019.02.004 30889383 PMC6428690

[44] Mengwasser KE Adeyemi RO Leng Y Choi MY Clairmont C D’Andrea AD. Genetic Screens Reveal FEN1 and APEX2 as BRCA2 Synthetic Lethal Targets. Mol Cell (2019) 73(5):885–99.e6. doi: 10.1016/j.molcel.2018.12.008 30686591 PMC6892393

[45] Dev H Chiang TW Lescale C de Krijger I Martin AG Pilger D. Shieldin Complex Promotes DNA End-Joining and Counters Homologous Recombination in BRCA1-Null Cells. Nat Cell Biol (2018) 20(8):954–65. doi: 10.1038/s41556-018-0140-1 PMC6145444 30022119

[46] Gogola E Duarte AA de Ruiter JR Wiegant WW Schmid JA de Bruijn R. Selective Loss of PARG Restores PARylation and Counteracts PARP Inhibitor-Mediated Synthetic Lethality. Cancer Cell (2018) 33(6):1078–93.e12. doi: 10.1016/j.ccell.2018.05.008 29894693

[47] Hamilton TC Young RC McKoy WM Grotzinger KR Green JA Chu EW. Characterization of a Human Ovarian Carcinoma Cell Line (NIH:OVCAR-3) With Androgen and Estrogen Receptors. Cancer Res (1983) 43(11):5379–89. 6604576

[48] Hills CA Kelland LR Abel G Siracky J Wilson AP Harrap KR. Biological Properties of Ten Human Ovarian Carcinoma Cell Lines: Calibration *In Vitro* Against Four Platinum Complexes. Br J Cancer (1989) 59(4):527–34. doi: 10.1038/bjc.1989.108 PMC2247140 2653399

[49] Yuan Y Kim WH Han HS Lee JH Park HS Chung JK. Establishment and Characterization of Human Ovarian Carcinoma Cell Lines. Gynecol Oncol (1997) 66(3):378–87. doi: 10.1006/gyno.1997.4785 9299249

[50] Stordal B Timms K Farrelly A Gallagher D Busschots S Renaud M. BRCA1/2 Mutation Analysis in 41 Ovarian Cell Lines Reveals Only One Functionally Deleterious BRCA1 Mutation. Mol Oncol (2013) 7(3):567–79. doi: 10.1016/j.molonc.2012.12.007 PMC4106023 23415752

[51] McGuire WP Hoskins WJ Brady MF Kucera PR Partridge EE Look KY. Cyclophosphamide and Cisplatin Compared With Paclitaxel and Cisplatin in Patients With Stage III and Stage IV Ovarian Cancer. N Engl J Med (1996) 334(1):1–6. doi: 10.1056/NEJM199601043340101 7494563

[52] Schilder RJ Hall L Monks A Handel LM Fornace AJ Jr. Ozols RF. Metallothionein Gene Expression and Resistance to Cisplatin in Human Ovarian Cancer. Int J Cancer (1990) 45(3):416–22. doi: 10.1002/ijc.2910450306 2307530

[53] Baloch T Lopez-Ozuna VM Wang Q Matanis E Kessous R Kogan L. Sequential Therapeutic Targeting of Ovarian Cancer Harboring Dysfunctional BRCA1. BMC Cancer (2019) 19(1):44. doi: 10.1186/s12885-018-5250-4 30630446 PMC6327434

[54] Franken NA Rodermond HM Stap J Haveman J van Bree C. Clonogenic Assay of Cells In Vitro. Nat Protoc (2006) 1(5):2315–9. doi: 10.1038/nprot.2006.339 17406473

[55] Chou TC. Drug Combination Studies and Their Synergy Quantification Using the Chou-Talalay Method. Cancer Res (2010) 70(2):440–6. doi: 10.1158/0008-5472.CAN-09-1947 20068163

[56] Yasmeen A Beauchamp MC Piura E Segal E Pollak M Gotlieb WH. Induction of Apoptosis by Metformin in Epithelial Ovarian Cancer: Involvement of the Bcl-2 Family Proteins. Gynecol Oncol (2011) 121(3):492–8. doi: 10.1016/j.ygyno.2011.02.021 21388661

[57] Ame JC Fouquerel E Gauthier LR Biard D Boussin FD Dantzer F. Radiation-Induced Mitotic Catastrophe in PARG-Deficient Cells. J Cell Sci (2009) 122(Pt 12):1990–2002. doi: 10.1242/jcs.039115 19454480

[58] Shirai H Fujimori H Gunji A Maeda D Hirai T Poetsch AR. Parg Deficiency Confers Radio-Sensitization Through Enhanced Cell Death in Mouse ES Cells Exposed to Various Forms of Ionizing Radiation. Biochem Biophys Res Commun (2013) 435(1):100–6. doi: 10.1016/j.bbrc.2013.04.048 23624507

[59] Fogh J T G. New Human Tumor Cell Lines. In: Human Tumor Cells In Vitro. 1975. Boston, MA, USA: Springer (1975). p. 115–59.

[60] Hamilton TC Young RC Ozols RF. Experimental Model Systems of Ovarian Cancer: Applications to the Design and Evaluation of New Treatment Approaches. Semin Oncol (1984) 11(3):285–98. 6385258

[61] Morgan MA Parsels LA Zhao L Parsels JD Davis MA Hassan MC. Mechanism of Radiosensitization by the Chk1/2 Inhibitor AZD7762 Involves Abrogation of the G2 Checkpoint and Inhibition of Homologous Recombinational DNA Repair. Cancer Res (2010) 70(12):4972–81. doi: 10.1158/0008-5472.CAN-09-3573 PMC2889008 20501833

